# Complete genome sequences of the *Serratia plymuthica* strains 3Rp8 and 3Re4-18, two rhizosphere bacteria with antagonistic activity towards fungal phytopathogens and plant growth promoting abilities

**DOI:** 10.1186/s40793-016-0185-3

**Published:** 2016-09-06

**Authors:** Eveline Adam, Henry Müller, Armin Erlacher, Gabriele Berg

**Affiliations:** Institute of Environmental Biotechnology, Graz University of Technology, Petersgasse 12, 8010 Graz, Austria

**Keywords:** *Serratia plymuthica*, Biocontrol, Plant growth promotion, Secretion systems, Antagonistic rhizosphere bacteria

## Abstract

The *Serratia plymuthica* strains 3Rp8 and 3Re4-18 are motile, Gram-negative, non-sporulating bacteria. Strain 3Rp8 was isolated from the rhizosphere of *Brassica napus* L. and strain 3Re4-18 from the endorhiza of *Solanum tuberosum* L. Studies have shown in vitro activity against the soil-borne fungi *Verticillium dahliae* Kleb., *Rhizoctonia solani* Kühn, and *Sclerotinia sclerotiorum*. Here, we announce and describe the complete genome sequence of *S. plymuthica* 3Rp8 consisting of a single circular chromosome of 5.5 Mb that encodes 4954 protein-coding and 108 RNA-only encoding genes and of *S. plymuthica* 3Re4-18 consisting of a single circular chromosome of 5.4 Mb that encodes 4845 protein-coding and 109 RNA-only encoding genes. The whole genome sequences and annotations are available in NCBI under the locus numbers CP012096 and CP012097, respectively. The genome analyses revealed genes putatively responsible for the promising plant growth promoting and biocontrol properties including predicting factors such as secretion systems, iron scavenging siderophores, chitinases, secreted proteases, glucanases and non-ribosomal peptide synthetases, as well as unique genomic islands.

## Introduction

*Serratia* species are well known for their potential as biocontrol agents with broad-spectrum antagonistic activities against common phytopathogens and their plant growth-promoting abilities. *Serratia plymuthica* 3Rp8 was isolated as an indigenous colonizer of oilseed rape (*Brassica napus* L.) rhizosphere and is an in vitro antagonist of the soil-borne fungal phytopathogens *Verticillium dahliae* Kleb., *Rhizoctonia solani* Kühn and *Sclerotinia sclerotiorum* [1] which can cause severe yield losses in a large number of different crops. Chitinase and protease activity were demonstrated by plate assays and the production of N-acylhomoserine lactones was detected using bioluminescent sensor plasmid pSB403 [1, 2]. *Serratia plymuthica* 3Re4-18 was isolated from the endorhiza of a potato plant (*Solanum tuberosum* L.) and was identified as the most effective isolate in an in vitro study screening potato-associated bacterial communities for antagonistic functions against plant pathogenic fungi [3]. Both strains were sequenced to augment current studies targeting novel biotechnological applications for seed and root treatment since the strains represent promising candidates for biological control. In this report, we summarize the complete genome sequences and annotations of *S. plymuthica* 3Rp8 and 3Re4-18 and describe their genomic properties. Analysis of the genomes of 3Rp8 and 3Re4-18 will provide a framework for further studies of their rhizosphere competence, biocontrol properties, and plant growth promoting activity. 3Rp8 and 3Re4-18 are deposited in the strain collection of antagonistic microorganisms at Graz University of Technology, Institute of Environmental Biotechnology, Austria.

## Organism information

### Classification and features

**Fig. 1:**
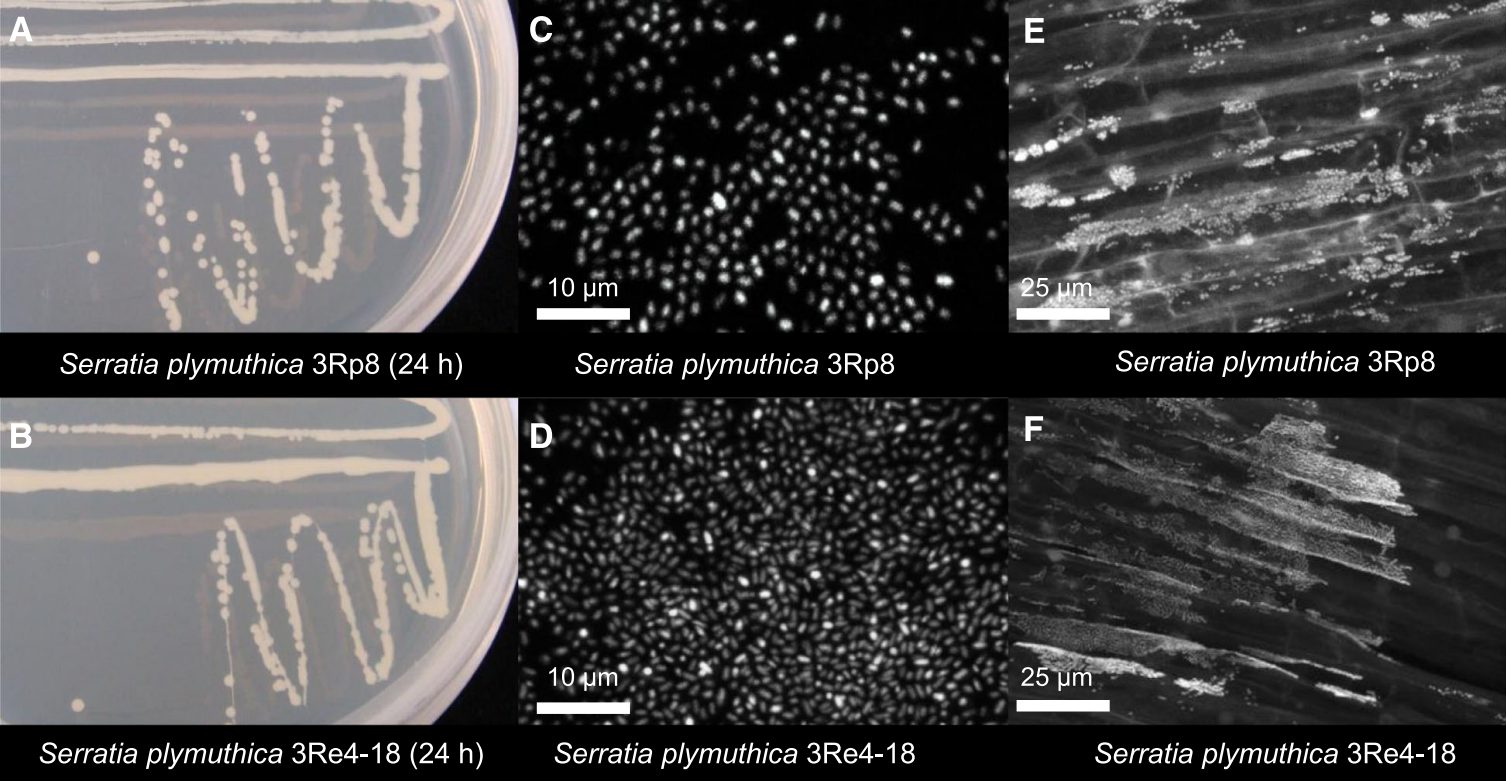
*S. plymuthica* 3Rp8 and 3Re4-18 on solid media and Confocal Laser Scanning Microscopy micrographs. **a**-**b** *S. plymuthica* 3Rp8 and 3Re4-18 grown on LB solid media after 24 h at 30 °C. Confocal Laser Scanning Microscopy micrographs: **c** and **d** show the cell morphology of pure cultures of 3Rp8 and 3Re4-18 after SYTO 9 green-fluorescent staining. **e**-**f** Fluorescence *in situ* hybridized 3Rp8 and 3Re4-18 colonizing the roots of young lettuce seedlings 1 week after inoculation in a gnotobiotic plant growth approach

*S. plymuthica* 3Rp8 and 3Re4-18 are motile, Gram-negative, non-sporulating *Enterobacteriaceae**.* Colonies appear yellow-beige opaque, domed and moderately mucoid with smooth margins on Luria-Bertani (LB) solid media and form colonies within 24 h at 20 °C (Fig. 1a-b). Both strains grow in standard complex media such as LB, potato dextrose agar (PDA), Waksman agar (WA) and nutrient agar (NA) [4] as well as in minimal medium such as Standard Succinate Medium (SSM). The standard growth temperature is at 30 °C, but both strains can replicate in liquid LB at 5 °C and at 40 °C as well. Both strains do not show a production of red pigments on the media mentioned above. The rod-shaped cells are approximately 0.5 μm in width and 2.0 μm in length (Fig. 1c-d).

3Rp8 was isolated from the roots of oilseed rape cultivar Express grown for a field trial in Braunschweig (Germany) in 1998 [1, 5]. 3Re4-18 was isolated from the endorhiza of an early senescent *Solanum tuberosum* L. cultivar Cilena at the experimental station of the Institute for Plant Diseases, Bonn University in Bonn-Poppelsdorf (Germany) in 2001 [3].

**Fig. 2:**
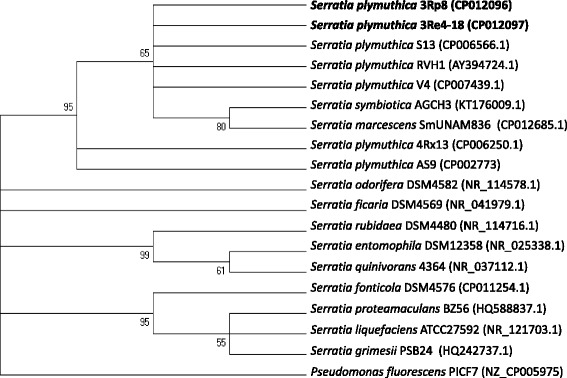
Maximum likelihood 16S rDNA phylogenetic tree indicating the phylogenetic relationship of sequenced isolates. The phylogenetic relationships inferred from the alignment of 1532 bp of 16S rDNA highlighting the positions of *S. plymuthica* 3Rp8 and 3Re4-18 relative to their closest *Serratia* strains for which 16S rDNA sequences are publicly available. A representative rhizosphere bacterium from the genera *Pseudomonas* was used as outgroup. The evolutionary history was inferred by using the Maximum Likelihood method based on the Tamura-Nei model [35]. The percentage of trees in which the associated taxa clustered in the bootstrap test (1000 replicates) is shown next to the branches [36]. Evolutionary analyses were conducted in MEGA7 [37]

**Fig. 3:**
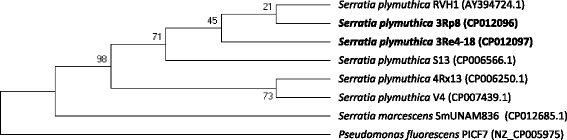
Maximum likelihood phylogenetic tree inferred from three housekeeping genes. The phylogenetic relationships inferred from the alignment of 8077 bp of concatenated DNA from three housekeeping genes highlighting the positions of *S. plymuthica* 3Rp8 and 3Re4-18 relative to their closest *Serratia* strains for which complete genomes are publicly available. A representative rhizosphere bacterium from the genera *Pseudomonas* was used as outgroup. For the construction of the tree, the protein-coding house-keeping genes *gyr*B (2420 bp), *rpo*P (4146 bp) and *nus*A (1511 bp) were concatenated and aligned. Then the evolutionary history was inferred by using the Maximum Likelihood method based on the Tamura-Nei model [35]. The percentage of trees in which the associated taxa clustered in the bootstrap test (1000 replicates) is shown next to the branches [36]. Evolutionary analyses were conducted in MEGA7 [37]

**Fig. 4:**
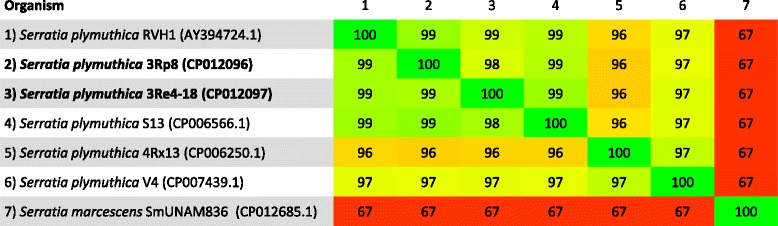
Phylogenomic overview using ANI data calculated from whole genome sequences. The heat-plot was compiled in Gegenees [6] and is based on a fragmented alignment using BLASTN made with settings 200/100 (accurate calculation). The cutoff threshold for non-conserved material was set to 30 %

Both bacterial strains are efficient colonizer of oilseed rape and cauliflower [4], lettuce and pumpkin roots (unpublished data) and do not cause any obvious negative effects to those hosts. Priming of oilseed rape and cauliflower seeds with the *S. plymuthica* 3Rp8 and 3Re4-18 strains had a significant PGP effect on the root weights of the oilseed rape seedlings [4]. Figure 1e-f shows 3Rp8 and 3Re4-18 colonizing the roots of young lettuce seedlings 1 week after inoculation in a gnotobiotic plant growth approach. The strains have natural resistance to Cefuroxime, Cefuroxime Axetil and Cefoxitin (minimal inhibitory concentration (MIC) > = 64 mg/L) as well as Fosfomycin (MIC > = 256 mg/L). Minimum Information about the Genome Sequences (MIGS) of *S. plymuthica* 3Rp8 and 3Re4-18 are summarized in Table 1, and their phylogenetic position is shown in Figs. 2 and 3. Average nucleotide identity (ANI) data were calculated with Gegenees [6] version 2.2.1 by using a fragmented all against all comparison. The data are illustrated as heat-plot in Fig. 4.

**Table 1:** Classification and general features of *Serratia plymuthica* 3Rp8 and 3Re4-18 according to the MIGS recommendations [20]

MIGS ID	Property	Term	Evidence code^a^
	Classification	Domain *Bacteria*	TAS [21]
		Phylum *Proteobacteria*	TAS [22]
		Class *Gammaproteobacteria*	TAS [23, 24]
		Order “*Enterobacteriales”*	TAS [25]
		Family *Enterobacteriaceae*	TAS [26–28]
		Genus *Serratia*	TAS [26, 29, 30]
		Species *Serratia plymuthica*	TAS [26, 31]
		Strain *Serratia plymuthica* 3Rp8	TAS [1]
		Strain *Serratia plymuthica* 3Re4-18	TAS [3]
	Gram stain	Gram-negative	TAS [30]
	Cell shape	Rod-shaped	IDA
	Motility	Motile	IDA
	Sporulation	Non-spore forming	IDA
	Temperature range	5-40 °C	IDA
	Optimum temperature	30 °C	IDA
	pH range; Optimum	5–9; 6	IDA
	Carbon source	Heterotrophic	IDA, TAS [1, 3, 4]
MIGS-6	Habitat	Root-associated	TAS [1, 3]
MIGS-6.3	Salinity	3Rp8 - 0.5 %-8 % NaCl (w/v) 3Re4-18 - 0.5 %-9 % NaCl (w/v)	IDA
MIGS-22	Oxygen requirement	Facultative anaerobe	TAS [30, 32]
MIGS-15	Biotic relationship	3Rp8 - Rhizospheric 3Re4-18 - Root endophytic	IDA, TAS [1] IDA, TAS [3]
MIGS-14	Pathogenicity	Non-pathogenic	NAS, TAS [30, 33]
MIGS-4	Geographic location	3Rp8 - North Germany 3Re4-18 - West Germany	TAS [1] TAS [3]
MIGS-5	Sample collection	3Rp8 - 1998 3Re4-18 - 2001	TAS [1] TAS [3]
MIGS-4.1	Latitude	3Rp8 - ~52.27 N 3Re4-18 - ~50.72 N	NAS
MIGS-4.2	Longitude	3Rp8 - ~10.57 E 3Re4-18 - ~7.09 E	NAS
MIGS-4.4	Altitude	3Rp8 - ~72 m.a.s.l. 3Re4-18 - ~63 m.a.s.l.	NAS

^a^Evidence codes - IDA: Inferred from Direct Assay; TAS: Traceable Author Statement (i.e., a direct report exists in the literature); NAS: Non-traceable Author Statement (i.e., not directly observed for the living, isolated sample, but based on a generally accepted property for the species, or anecdotal evidence). These evidence codes are from the Gene Ontology project [34]

## Genome sequencing information

### Genome project history

The strains *S. plymuthica* 3Rp8 and 3Re4-18 were selected for sequencing due to their in vitro activity against *V. dahliae* and *R. solani*, their production of hydrolytic enzymes and their root-associated lifestyle on plants [1, 3, 4]. The sequence data will help to reveal genetic features responsible for their plant growth promoting effects and their ability to protect seeds against fungal threats during germination. The genome project is deposited in the NCBI BioProject database under ID 289082 with the Biosample UIDs 3841799 and 3841798, respectively. The finished genome sequences are deposited in GenBank under the accession numbers CP012096 and CP012097, respectively. A summary of the project information is shown in Table 2.

**Table 2:** Project information

MIGS ID	Property	Term
MIGS 31	Finishing quality	Finished
MIGS 28	Libraries used	PacBio RS libraries with inserts of 8 to 20 kb
MIGS 29	Sequencing platforms	PacBio RS II
MIGS 31.2	Fold coverage	3Rp8 - 81 x 3Re4-18 - 110 x
MIGS 30	Assemblers	Celera Assembler + Hierarchical genome assembly process v. 2.2.0
MIGS 32	Gene calling method	NCBI Prokaryotic Genome Annotation Pipeline, Glimmer gene prediction
	Locus Tag	3Rp8 - ADP72 3Re4-18 - ADP73
	Genbank ID	3Rp8 - CP012096 3Re4-18 - CP012097
	GenBank Date of Release	June 15, 2016
	GOLD ID	3Rp8 - Gp0137065 3Re4-18 - Gp0131532
	BIOPROJECT	PRJNA289082
MIGS 13	Source Material Identifier	3Rp8 - SAMN03841799 3Re4-18 - SAMN03841798
	Project relevance	Agricultural, Environmental

### Growth conditions and genomic DNA preparation

3Rp8 and 3Re4-18 were grown in 50 ml of nutrient broth II (NB II) (Sifin, Berlin, Germany) medium and incubated for 20 h at 30 °C. 0.5 ml was then centrifuged at 2500 x g for 5 min at 4 °C and genomic DNA was extracted using the MasterPure DNA purification kit (Epicentre, Madison, WI, USA). DNA quality and quantity were checked by agarose gel electrophoresis and spectrophotometry using a UV-Vis spectrophotometer (NanoDrop 2000c, Thermo Fisher Scientific, Waltham, MA USA). Total genomic DNA of 3Rp8 (50.7 μg; 0.8 μg μL^-1^) and of 3Re4-18 (102.8 μg; 1.7 μg μL^-1^) was sent on dry ice to the sequencing service.

### Genome sequencing and assembly

PacBio RS libraries with inserts of 8 to 20 kb were constructed and sequenced at GATC Biotech (Konstanz, Germany) using single molecule, real-time (SMRT) sequencing. Assemblies were completed with the Hierarchical Genome Assembly Process v. 2.2.0 (HGAP) algorithm implemented in the PacBio SMRT Analysis software (Pacific Biosciences, Menlo Park, CA, USA). The assembly of the 3Rp8 genome was based on 119,662 quality reads with a mean length of 4581 bp resulting in a single circular chromosome consisting of 5,546,041 bp with 81-fold overall coverage. For assembling the genome of 3Re4-18, 127,834 quality reads with a mean length of 5358 bp were used resulting in a single circular chromosome of 5,439,574 bp with 110-fold overall coverage.

### Genome annotation

Automatic annotation was performed using the NCBI Prokaryotic Genome Annotation Pipeline (released 2013). Additional annotation for using the automated assignment of clusters of orthologous groups (COG)-functions to protein-coding genes was completed on the BASys Web server using Glimmer gene prediction [7–9]. Prediction of Pfam domains, signal peptides and transmembrane helices were calculated using BASys Web Server [7–9], SignalP [10, 11] and TMHMM [12, 13], respectively.

## Genome properties

**Fig. 5:**
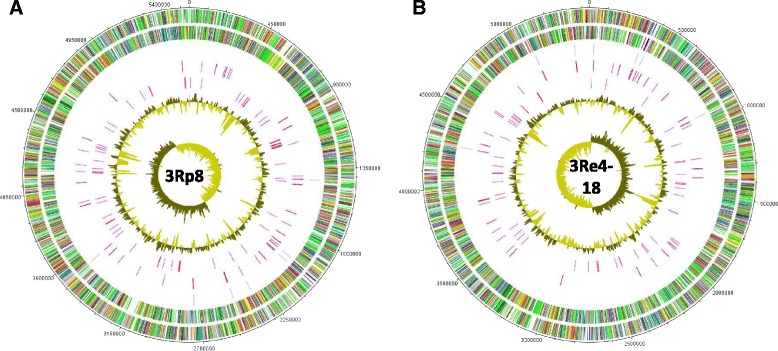
Graphical map of the chromosome of 3Rp8 (**a**) and 3Re4-18 (**b**). The outer scale is marked every 10 kb. Circles range from 1 (outer circle) to 7 (inner circle). Circle 1 and 2, ORFs encoded by leading and lagging strand respectively, with color code for functions: salmon, translation, ribosomal structure and biogenesis; aquamarine, RNA processing and modification; light blue, transcription; cyan, DNA replication, recombination and repair; tan, chromatin structure and dynamics; turquoise, cell division; dark orange, defense mechanisms; deep pink, post-translational modification, protein turnover and chaperones; dark olive green, cell envelope biogenesis; purple, cell motility and secretion; lavender, intracellular trafficking, secretion, and vesicular transport; forest green, inorganic ion transport and metabolism; pink, signal transduction; red, energy production; sienna, carbohydrate transport and metabolism; yellow, amino acid transport; orange, nucleotide transport and metabolism; gold, co-enzyme transport and metabolism; cornflower blue, lipid metabolism; blue, secondary metabolites, transport and catabolism; gray, general function prediction only; yellow green, unknown function; black, function unclassified or unknown. Circle 3 and 4, distributions of tRNA genes and rrn operons respectively. Circle 5, distribution of pseudogenes. Circle 6 and 7, G + C content and GC skew (G-C/G + C) respectively

The genome of *S. plymuthica* strain 3Rp8 is composed of one circular chromosome consisting of 5,546,041 bp with an average GC content of 56.07 % (Table 3 and Fig. 5a). Among the 5130 predicted genes, 4954 (96.57 %) were identified as protein coding genes, 68 (1.33 %) were designated as pseudo genes, 22 (0.43 %) as rRNAs, 85 (1.66 %) as tRNAs and one (0.02 %) as ncRNA. 21 (0.41 %) genes were frameshifted.

**Table 3:** Genome statistics

	3Rp8		3Re4-18	
Attribute	Value	% of Total^a^	Value	% of Total^a^
Genome size (bp)	5,546,041	100.00	5,439,574	100.00
DNA coding (bp)	4,745,098	85.56	4,683,982	86.11
DNA G + C (bp)	3,109,696	56.07	3,058,992	56.24
DNA scaffolds	1	-	1	-
Total genes	5130	100.00	5005	100.00
Protein coding genes	4954	96.57	4845	96.80
RNA genes	108	2.11	109	2.18
Pseudo genes	68	1.33	51	1.02
Genes in internal clusters	NA	-	NA	-
Genes with function prediction	4278	83.39	4239	84.70
Genes assigned to COGs	4077	79.47	4017	80.26
Genes with Pfam domains	3829	74.64	3780	75.52
Genes with signal peptides	499	9.73	489	9.77
Genes with transmembrane helices	1239	24.15	1213	24.24
CRISPR repeats	0	0	0	0

^a^The total is based on either the size of the genome in base pairs or the total number of genes in the annotated genome

The genome of *S. plymuthica* strain 3Re4-18 is composed of one circular chromosome of 5,439,574 bp with an average GC content of 56.24 % (Table 3 and Fig. 5b). Among the 5005 predicted genes, 4845 (96.80 %) were identified as protein coding genes, 51 (1.02 %) were designated as pseudo genes, 22 (0.44 %) as rRNAs, 86 (1.72 %) as tRNAs and one (0.02 %) as ncRNA. 19 (0.38 %) genes were frameshifted.

The GC contents of both strains are similar to that of other *S. plymuthica* strains. The classification of CDSs into functional categories according to the COG database [14, 15] is summarized in Table 4 on BASys gene prediction.

**Table 4:** Number of genes associated with general COG functional categories

Code	3Rp8		3Re4-18		Description
	Value	%age	Value	%age	
J	169	2.90	167	2.97	Translation, ribosomal structure and biogenesis
A	1	0.02	1	0.02	RNA processing and modification
K	441	7.57	445	7.92	Transcription
L	170	2.92	152	2.70	Replication, recombination and repair
B	1	0.02	1	0.02	Chromatin structure and dynamics
D	27	0.46	28	0.50	Cell cycle control, cell division, chromosome partitioning
V	58	1.00	56	1.00	Defense mechanisms
T	141	2.42	146	2.60	Signal transduction mechanisms
M	256	4.40	256	4.55	Cell wall/membrane biogenesis
N	99	1.70	90	1.60	Cell motility
U	54	0.93	49	0.87	Intracellular trafficking and secretion
O	153	2.63	148	2.63	Posttranslational modification, protein turnover, chaperones
C	261	4.48	259	4.61	Energy production and conversion
G	412	7.08	406	7.22	Carbohydrate transport and metabolism
E	442	7.59	433	7.70	Amino acid transport and metabolism
F	89	1.53	90	1.60	Nucleotide transport and metabolism
H	144	2.47	145	2.58	Coenzyme transport and metabolism
I	150	2.58	138	2.45	Lipid transport and metabolism
P	246	4.22	246	4.38	Inorganic ion transport and metabolism
Q	96	1.65	91	1.62	Secondary metabolites biosynthesis, transport and catabolism
R	383	6.58	385	6.85	General function prediction only
S	284	4.88	285	5.07	Function unknown
-	1746	29.98	1605	28.55	Not in COGs

The percentage is based on the total number of protein coding genes in the genome based on BASys gene prediction [7–9]

## Insights from the genome sequence

Both strains share a collection of genes that may be important contributors to biological control with other *S. plymuthica* strains already published, like genes annotated as secretion systems, iron scavenging siderophores (locus tags ADP72_19185, ADP73_16995), chitinases (e.g. locus tags ADP72_04805, ADP73_00825), secreted proteases (e.g. locus tags ADP72_11930, ADP73_24375), glucanases (e.g. locus tags ADP72_10355, ADP73_00890) and non-ribosomal peptide synthetases (e.g. locus tags ADP72_05100, ADP73_05800). Additionally, genes predicting plant growth promotion, like spermidine synthases (e.g. locus tags ADP72_15170, ADP73_11985), indole-3-pyruvate decarboxylases (locus tags ADP72_18190, ADP73_17980) or diacetyl-reductase (locus tags ADP72_19475, ADP73_16745) were detected. Unique genomic islands were identified in both strains with IslandViewer 3 software [16–18]. In 3Rp8 coding regions containing high similarities on DNA-level with a region in *Photorhabdus luminescens* TT01 [19] as well as a region annotated as type IV/VI secretion system were found. In 3Re4-18 unique coding regions for proteins related to type VI secretion systems as well as other islands with putatively phage origin were detected.

## Conclusions

Here, we announce the complete genome sequences of *Serratia plymuthica* 3Rp8 and 3Re4-18, two enterobacteria that were originally isolated in Germany from oilseed rape rhizosphere and from endorhiza of potato, respectively. Both strains were selected for sequencing based on their ability to control soil-borne plant-pathogenic fungi. Such properties likely have origins in a repertoire of genes probably involved in fungal cell wall degradation expressed by chitinases, proteases or non-ribosomal peptide synthetases. They also share a collection of genes known to be responsible for specific PGP features and both carry unique genomic islands with interesting genes for agricultural applications. Further functional studies and comparative genomics with related isolates will greatly enhance the understanding of biocontrol and PGP features.

## Abbreviations

MIC: Minimal inhibitory concentration
PGP: Plant growth promoting
SMRT: Single molecule, real-time
